# Prevalence of Teenage Pregnancy in A Community Hospital of Rural Nepal: A Cross-sectional Study

**DOI:** 10.31729/jnma.4083

**Published:** 2019-06-30

**Authors:** Muna Maharjan, Niresh Thapa, Narayani Maharjan, Pabita Rai, Prakash Pun, Marcia A Petrini, Jiong Yang

**Affiliations:** 1 HOPE School of Nursing, Zhongnan Hospital of Wuhan University, Wuhan, Hubei, China; 2 Department of Gynecological Oncology, Zhongnan Hospital of Wuhan University, Hubei Cancer Clinical Study Center, Hubei Key Laboratory of Tumor Biological Behaviors, Wuhan, P.R. China; 3 Department of General Practice and Emergency Medicine, Karnali Academy of Health Sciences, Jumla, Nepal; 4 Department of Clinical Laboratory Science, Zhongnan Hospital of Wuhan University, Wuhan, Hubei, China; 5 Okhaldhunga Community Hospital, Okhaldhunga, Nepal; 6 Care and Cure Hospital, Kapilvastu, Nepal; 7 Faculty of Nursing, Chiang Mai University, Chiang Mai, Thailand; 8 Department of Respiratory Medicine, Zhongnan Hospital of Wuhan University, Wuhan, China

**Keywords:** *pregnancy*, *rural hospital*, *teenage pregnancy*

## Abstract

**Introduction:**

Teenage pregnancy is a public health concern. Maternal and neonatal health outcomes are negatively impacted in teenage pregnancy. The objective of the study is to find the prevalence of teenage pregnancy in a community hospital of rural Nepal.

**Methods:**

A descriptive cross-sectional study was conducted at Okhaldhunga Community Hospital, Okhaldhunga, Nepal. Ethical approval was taken from the Institutional Review Committee of the hospital. Data were retrieved from July 2007 to July 2017 from the hospital record books. The total of 7054 records of deliveries were reviewed from the hospital records and whole sampling was done. Subgroup analysis was done on basis of age, ethnicity, gravida, para, period of gestation, mode of delivery, maternal or neonatal complications and birth weight. Data entry was done in Microsoft Excel and point estimate at 95% CI was calculated along with frequancy and proportion for binary data.

**Results:**

The total of 7054 deliveries were conducted in ten years among which 2050 (29.06%) were teenage deliveries at the 95% CI (28.52 to 29.06). The highest percentage of teenage delivery was found among Janajati ethnicity of 1056 (53.3%). Amongst teenage delivery, a significant tear was found in 157 (7.9%) as a maternal complication. Perinatal deaths were found in 27 (1.4%).

**Conclusions:**

The trend of teenage pregnancy remains almost same over ten years in the Okhaldhunga Community Hospital. The overall prevalence of teenage delivery is higher than the national figure. Low birth weight babies, premature delivery, perineal and cervical tears were the common complications. Further health education and awareness programs might help to reduce the teenage pregnancy rate.

## INTRODUCTION

Teenage pregnancy, pregnancy within 19 years of age, is one of the most critical social and public health problems both in developed and developing countries. Worldwide, around 16 million girls between the ages of 15 and 19, and two million girls under age 15 become pregnant every year.^1^

In Nepal, adolescents aged 10-19 years comprise of 6.38 million of the total population of 28.5 million.^2^ From 1996 to 2011, the adolescent pregnancy rate decreased from 24% to 17%, but the median age at first pregnancy remained 16.2 years.^3,4^ In Nepal, the legal minimum age of marriage for a woman is 20.^5^ However, child marriage is still prevalent, and 29% of women get married before they turn 20.^6^ Teenage pregnancy has an increased risk of maternal and neonatal morbidity and mortality.^1^

Therefore, the study aims to find the prevalence of teenage pregnancy in a community hospital of rural part of Nepal.

## METHODS

A descriptive cross-sectional study was approved by the Institutional Review Committee of Okhaldhunga Community Hospital (OCH) run by United Mission to Nepal (UMN). The total of 7054 records of deliveries were reviewed from the hospital records and whole sampling was done and datas were retrieved from July 2007 to July 2017 from the hospital record books of Okhaldhunga Community Hospital. The inclusion criteria were female who had delivered their baby in OCH in between the study period. Women who delivered baby outside of the hospital and who had incomplete datas were excluded from the study.

Data entry was done in Microsoft Excel and point estimate at 95% CI was calculated along with frequancy and proportion for binary data. Subgroup analysis was done based on demographic information such as age, ethnicity, gravida, para, the period of gestation, mode of delivery, maternal or neonatal complications and birth weight.

Low birth weight (LBW) baby was defined as a weight of a baby below 2500 gm at birth, and prematurity as delivery at a gestational age of fewer than 37 weeks of the period. Late fetal death or stillbirth was defined as death at >28 weeks of gestation. Neonatal death was defined as death occurring before 28 days of life. Gestational age was calculated based on the last menstrual period or using ultrasound.^7^

## RESULTS

The total of 7054 deliveries were conducted in Okhaldhunga Community Hospital from July 2007 to July 2017, among them, 2050 (29.06%) were teenage deliveries at 95% CI (28.52 to 29.06). The women with age less than 17 years were 271 (13.2%) and 1819 years were 1779 (86.8%). Majority of the women, 1056 (53.3%), belong to the Janajati ethnicity. Similarly, 1596 (91.3%) were primigravida, and 255 (12.9%) had a complicated delivery, which includes cesarean section, vacuum and forceps deliveries (Table 1).

**Table 1.** Characteristics of the participants.

Variables	n (%)
Total deliveries	7054
Teenage	2050 (29.06)
>20 years	5004 (70.94)
Age (years)	
<17	271 (13.2)
18-19	1779 (86.8)
Mean age	18.79 ± 1.13
Ethnicity	
Janajati	1056 (53.3)
Brahmin	343 (17.3)
Chhetri	310 (15.6)
Dalit	252 (12.7)
Others	32 (1.1)
Gravida	
Primigravida	1596 (91.3)
2 or more	153 (7.1)
Para	
0	1166 (92.5)
>1	94 (7.4)
Abortion	
0	1235 (98.0)
>1	25 (2.0)
Mode of delivery	
Normal	1728 (87.1)
Complicated	255 (12.9)

Among 1741 women, 84 (4.8%) had preterm delivery whereas, 309 (17.7%) were overdue. Similarly, 347 (17.9%) delivered baby less than 2500 gm birth weight (Table 2).

**Table 2.** Teenage delivery outcome.

Variables	n (%)
Period of Gestation (n=1741)	
<36 weeks	84 (4.8)
37 – 40 weeks	1348 (77.4)
> 40 weeks	309 (17.7)
Baby sex (n = 1945)	
Male	963 (49.5)
Female	982 (50.5)
Baby weight (n = 1943)	
<2500 gm	347 (17.9)
2500 – 4000 gm	1584 (81.5)
>4000 gm	12 (0.6)

**Figure 1.**
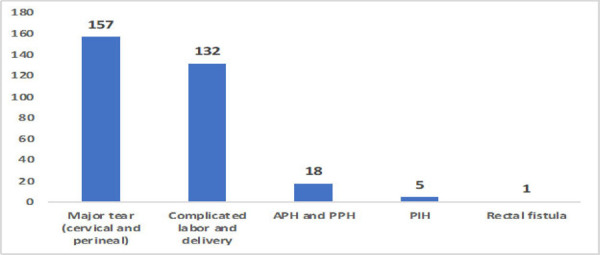
Maternal complications in teenage pregnancy.

Maternal complications among 1983 teenage deliveries with the highest frequency of a significant tear (cervical tear and perineal tear of the second degree or more) were 157 (7.9%), complicated labor and delivery was 132 (6.7%), antepartum and postpartum hemorrhage were 18 (0.9%) (Figure 1).

Among neonatal complications of 1983 teenage deliveries, perinatal death was 27 (1.4%), and asphyxia was 22 (1.1%) followed by premature birth.

Ten year trend of cesarean section among the women more than 20 years of age and teenage girls shows the highest rate of cesarean deliveries observed in the year of 2010/11 among teenage girls and 2011/12 among women aged more than 20 years. However, the overall cesarean section rate was around 12% (Figure 3).

**Figure 2.**
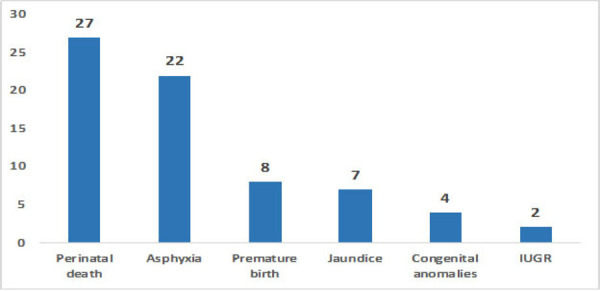
Neonatal complications in teenage pregnancy.

**Figure 3.**
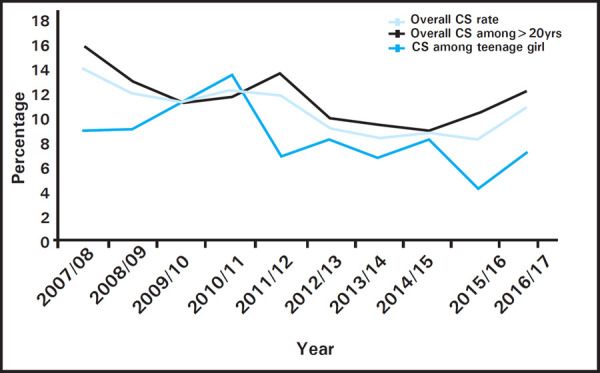
Trend of cesarean section.

**Figure 4.**
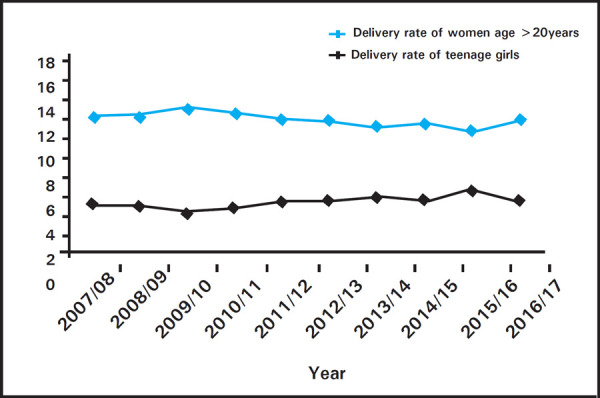
Trend of teenage delivery.

The trend of teenage delivery and delivery of women age 20 or above seems almost parallel (Figure 4).

## DISCUSSION

The present study showed the trend of deliveries over ten years, which reveals the prevalence of teenage deliveries of more than 29%. The number of hospital deliveries has been increasing since the 2007/08 to 2016/17, but the trend of teenage pregnancy remains the same. Teenage pregnancy is a global issue with the highest adolescent birth rates occurring in middle and low-income countries.^8^ As reported by Acharya et al., within South Asia, the recorded teenage pregnancy rate is highest in Bangladesh 35% followed by Nepal 21% and India 21%.^9^ There are higher proportions of adolescent pregnancies in South Asian countries because of the common practice of early marriage and social expectation to have a child soon after marriage.^10,11^ Choe et al. and Adhikari et al. also concluded that early marriage and sexual debut were associated with greater risk of adolescent pregnancy in Nepal.^10,12^

Teenage pregnancy is one of the risk factors for maternal and neonatal morbidity, and mortality.^13^ The prevalence of adolescent pregnancy is significantly higher in women from lower social classes compared to those from higher classes. This review also suggested that unfavorable socioeconomic conditions experienced by the people in community and family lead to high teenage birth.^14^ Similarly, religion and ethnicity can be a marker for differing sexual practices, use of family planning and the use of abortion.^6^ In Nepal, ethnicity influences adolescent pregnancy.^15^ Our study revealed that teenage pregnancy among women from the Janajati ethnic background accounts for 53.3%, Brahmin 17.3%, Chhetri 15.6%, and Dalit 12.7%. This finding contradicts with the findings from other studies in Nepal, which reported that women from the lower ethnic background (Dalit and Madhesi) reported significantly higher adolescent pregnancy than higher ethnic background (Brahmin and Chhetri).^16^ This variation might be because of the geographical location where most of the population belongs to the Janajati (Rai, Magar, Tamang, Sherpa, Newar) with 50.20%, Brahmin 39.61%, and Dalit only 9.17%.^17^

In regards to maternal outcome, the majority of the teenage mothers were from age 18-19 years, and most of the cases were primigravida, which is similar to the other study findings.^18^ Regarding the mode of deliveries, 11.9% was cesarean delivery. This finding differs with the study conducted in other parts of Nepal that shows the cesarean delivery rate of up to 45%. The variance could be because of the other studies conducted in an urban teaching hospital.^18,19^ According to the Nepal Demographic Health Survey 2016, overall cesarean section deliveries were twice as prevalent in urban areas (12%) than in rural areas (6%).^20^ Mostly observed maternal complications were a cervical and perineal tear (7.9%), complicated labor and delivery (6.7%), antepartum and postpartum hemorrhage (0.9%), pregnancy-induced hypertension (0.3%) and rectal fistula (0.1%). These findings are almost similar to the results of other studies.^18,21^

The present study shows, only 4.8% were premature delivery, and 17.9% were low birth weight, which are adverse neonatal outcomes of teenage pregnancy. The findings from studies conducted by Rita et al., and Subedi et al., also reported the incidence of LBW with 12.5% to 13.4% respectively and associated with young maternal age.^18,22^ Perinatal death, asphyxia, premature birth, pathological jaundice, congenital anomalies, and intrauterine growth retardation were the neonatal complications found in this study.

There were some limitations to this study. The study with retrieved ten-year data from the hospital register was conducted. Therefore, the ability to collect all the required information due to the incomplete data was a barrier to comprehensive information. Moreover, it was a single hospital-based study, so the findings of this study cannot be generalized.

## CONCLUSIONS

The overall prevalence of teenage delivery is higher than the national figure. The trends of teenage pregnancy remain almost the same over the 10 years at the Okhaldhunga Community Hospital. Premature delivery, low birth weight baby, significant perineal and cervical tears were common complications. Further health education and awareness programs might help to reduce the teenage pregnancy rate.
